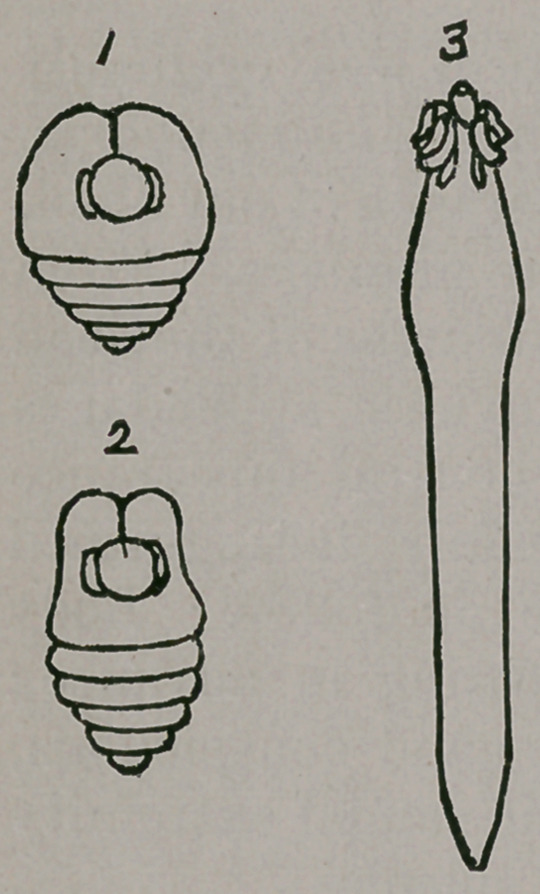# Parasites of the Shad and Herring

**Published:** 1888-07

**Authors:** Joseph Leidy

**Affiliations:** Director Department of Biology, University of Pennsylvania


VOL. IX. JULY, 1888. No. 3

### ORIGINAL COMMUNICATIONS.

### ART. XV.—PARASITES OF THE SHAD AND HERRING.

BY JOSEPH LEIDY, M.D., LL.D.

*Director Department of Biology, University of Pennsylvania.*

With the view of ascertaining the parasites of the Shad, *Alosa sapidissima*, I examined the entrails of fifty fish brought to our market from the rivers of the South and from the Delaware. Most of them were found to be infested with two nematoid worms, the *Agamonema capsularia*, Diesing, and the *Ascaris adunca*, Rudolphi, and all of them with the larva or scolex of a cestoid worm, which I propose to distinguish as *Gymnoscolex picta*. I also examined half a dozen of the closely allied fish, the common Herring, *Clupea harengus*, in which I also found the *Agamonema capsularia* and *Gymnoscolex picta*, but not the *Ascaris adunca*.

AGAMONEMA CAPSULARIA, Diesing.— This worm, known only in the immature sexual condition, is a frequent and common parasite of many fishes. Called by Linnæus the *Gordius marinus*, it has been described under various other names by different authors. Observed in Europe as a parasite of the Herring, it has also been there noticed in the Salmon, Mackerel, Cod, Turbot, Halibut and other fishes. In our Herring it appears to be a constant parasite, sometimes few and often in considerable numbers. It occupies the abdominal cavity among the viscera usually encysted in the peritoneum about the stomach and intestine and especially the pyloric appendages and less frequently on the liver and roes. It often forms flat and close spiral coils, lying on the viscera, or appended to them. Frequently it is observed free and incessantly wriggling ; but in this condition I suspect the worm has escaped from its cyst after the death of the Herring.

The Agamonema is also a common parasite of the Shad, and though usually occurring in small numbers, appears to be as constant as in the Herring. Mostly, too, it is larger than in the latter, and is found in the same positions and conditions. It is most frequently observed in conspicuous coils, appended to the viscera, and especially to the cœcal extremity of the stomach. It was found in every Shad examined, from three to a dozen or more.

The characters of the Agamonema of the Herring were as follows : Body slender, most tapering in advance, translucent white, and often with the intestines brownish, but in others white. Head rounded, truncate and bordered by conical papillæ, with the mouth unarmed, but furnished to one side with a minute conical spine attached by a broad base. Tail short, conical, incurved, blunt, but terminating in a minute mucro.

Length, 10 to 18 m m ; thickness, 0.25 to 0.375 m m ; tail, 0.12 to 0.24 m m long.

The œsophagus is long and cylindrical and is defined from the intestine by a marked constriction. The intestine has a long translucent diverticulum directed backward from its commencement and another more opaque directed forward along the opposite side of the œsophagus.

The Agamonema of the Shad ranged from 15 to 25 m m, by 0.3 and 0.5 to 0.625 m m, in thickness, with the tail 0.125 to 0.25 m m. In the smaller, translucent individuals the alimentary canal appeared to be like that in the

Agamonema of the Herring, but in the largest individuals I am uncertain whether the condition is the same.

The number of papillæ to the head of Agamonema appears to be three, but of this I could not satisfy myself. Wedl describes the Agamonema of the Mackerel as having the mouth armed with a conical tooth, which is capable of being protruded and retracted within a sheath. (Sitzungsb. Akad. Wiss. 1855, 18, Taf. III., Fig. 24.) In the worms under examination, I could see no such tooth, but to one side of the mouth, as above described, a little conical spine fixed by a spreading base, and commonly projecting obliquely forward and outward.

ASCARIS ADUNCA, Rudolphi.—This worm was originally described as a parasite of the European Shad, *Alosa vulgaris.* It is a frequent though not a constant parasite of our Shad, and commonly appears to occur few in numbers. From two to half a dozen were found in three-fourths of the Shad examined. It occurs in the intestine, pyloric appendages and less frequently in the stomach. In general appearance it resembles the Agamonema, but is readily distinguished by its large lips. Its characters are as follow: Body cylindrical, thickest posteriorly and tapering in advance; caudal extremity incurved. Lips large and conspicuous, tail short, conical, acute. Male with the caudal extremity spirally inrolled; with a pair of curved spicules.

Female 12 to 40 m m long; 0.3 to 1.125 m m thick; tail 0.125 to 0.25 m m long. Male, 20 to 30 m m long, 0.45 to 0.55 m m thick. In the smaller individuals the body is more uniformly cylindrical or less tapering in advance. This worm I formerly confounded with its associate Agamonema (Proc. Acad. Nat. Sci. Phila., 1856, 55).

GYMNOSCOLEX PICTA.—In all the Shad and Herring subjected to examination there occurred a larva or scolex of a cestoid worm, which, though closely resembling the *Scolex polymorphus*, I suspect to be different, and have therefore given it another name. The latter species is attributed to numerous marine fishes of European seas, but among them neither the Shad nor Herring have been indicated. *Scolex picta* appears to occur almost constantly in our Shad and

Herring. It was found along the course of the intestine, but especially in the pyloric portion and in its appendages. Commonly, not very numerous, sometimes there were but few, but generally from about a dozen to fifty and upwards. In the abundant mucus of the viscera, they appear as white granules, about the size of ordinary sand grains. Their characters are as follow : In the quiescent condition with the head withdrawn into the body ; spheroid, ovoid, ovate, or cordate ; in front rounded or truncate and more or less emarginate or projecting in a bipapillate manner ; posterior third conical, obtuse or sub-acute, and annularly rugose.

1

2

3

In the active condition with the head projected, clavate, elongating and becoming linear behind with the disappearance of the annular rugæ. Head provided with four hemispherical bothria and a central, spheroidal, cup-like rostellum, and with a bright red pigment streak on each side.

Measurements. In the quiescent condition, 0.5 to 0.625 m m diameter, or 0.5 to 0.75 and 0.875 m m long, by 0.375 to 0.5 m m broad. Elongating without projection of the head to 1.125 m m long, by 0.375 m m broad ; with projected head to 1.5, 1.75, 2, and 2.5 m m long, by 0.25 and 0.2 m m broad. Bothria 0.15 diameter ; central rostellum 0.1 m m diameter.

The scolex, when in motion with the head retracted, elongates and shortens and narrows and widens proportionately. In contraction, the posterior portion becomes more or less annularly constricted. In greatest activity, the head is protruded, and the body greatly elongated, and alternately shortens and elongates. With elongation the annular appearance of the posterior part entirely disappears. The rostellum and bothria are incessantly protruded and retracted, and become more or less oval in form. In the quiescent state by transmitted light, the head appears as a nearly central clearer spot embraced at the sides by the pigment streaks. When the bothria are protruded, the latter are situated posterior to them.

The body of the scolex is filled with the usual oval, clear, sharply defined corpuscles. Under moderate pressure the water-vascular system is brought into view. This appears as a tortuous vessel, proceeding forward on each side of the body from a little terminal caudal vesicle, returning from the head where it forms an expanded loop on each side of the rostellum.

With the scolex as described, there were found some smaller individuals having the same characters but without the conspicuous pigment streak.

The scolex of the Shad and Herring has a near resemblance to the *Scolex polymorphus*, Rudolphi, and it may, perhaps, prove to be the same. This species is described as having the bothria divided by a transverse partition, which is not the case in the scolex under consideration. Van Beneden, however, represents forms of the *S. polymorphus* of the Turbot, without the division of the bothria (Mem. Acad. de Belgique, XXV. pl. I., Figs 1—3).

The mature tape worm of the scolex of the Shad and Herring is yet unknown and no doubt has for its host some animal that appreciates these fishes for food quite as much as man does.

The accompanying figures, 1, 2, 3, represent *Scolex picta* magnified forty diameters; figures, 1, 2, in the quiescent condition with the head retracted; figure 3, in the active condition, elongated and with the head protruded.

## Figures and Tables

**Figure f1:**